# Bringing Real-Time Geospatial Precision to HIV Surveillance Through Smartphones: Feasibility Study

**DOI:** 10.2196/11203

**Published:** 2018-08-07

**Authors:** Alain Placide Nsabimana, Bernard Uzabakiriho, Daniel M Kagabo, Jerome Nduwayo, Qinyouen Fu, Allison Eng, Joshua Hughes, Samuel K Sia

**Affiliations:** ^1^ Junco Labs Kigali Rwanda; ^2^ Junco Labs New York, NY United States

**Keywords:** HIV surveillance, smartphones, mobile phones, geospatial data

## Abstract

**Background:**

Precise measurements of HIV incidences at community level can help mount a more effective public health response, but the most reliable methods currently require labor-intensive population surveys. Novel mobile phone technologies are being tested for adherence to medical appointments and antiretroviral therapy, but using them to track HIV test results with automatically generated geospatial coordinates has not been widely tested.

**Objective:**

We customized a portable reader for interpreting the results of HIV lateral flow tests and developed a mobile phone app to track HIV test results in urban and rural locations in Rwanda. The objective was to assess the feasibility of this technology to collect front line HIV test results in real time and with geospatial context to help measure HIV incidences and improve epidemiological surveillance.

**Methods:**

Twenty health care workers used the technology to track the test results of 2190 patients across 3 hospital sites (2 urban sites in Kigali and a rural site in the Western Province of Rwanda). Mobile phones for less than US $70 each were used. The mobile phone app to record HIV test results could take place without internet connectivity with uploading of results to the cloud taking place later with internet.

**Results:**

A total of 91.51% (2004/2190) of HIV test results could be tracked in real time on an online dashboard with geographical resolution down to street level. Out of the 20 health care workers, 14 (70%) would recommend the lateral flow reader, and 100% would recommend the mobile phone app.

**Conclusions:**

Smartphones have the potential to simplify the input of HIV test results with geospatial context and in real time to improve public health surveillance of HIV.

## Introduction

For the HIV/AIDS epidemic to be curtailed in a sustainable fashion, it will be critical to increase diagnosis, awareness, and tracking of HIV infections among the hardest hit, resource-constrained countries. Precise measurements of HIV incidences at a subnational level are instrumental in mounting an effective global response [1], but the most reliable methods currently require labor-intensive population surveys.

For HIV diagnostics, HIV rapid tests (which use lateral flow test technology) are widely used for primary screening. These tests are low cost, readily available, and can be performed in field settings, but they have shown lower specificity and sensitivity during field conditions as compared to laboratory evaluations, suggesting that there may be user variability in performing and reading the test results. Furthermore, test results are currently first entered by hand into a book and later transcribed into a computer. This process can introduce data entry errors and slows availability of the data for use by health care providers and officials. There exists an opportunity, using the latest technologies in mobile devices, to accurately record HIV test results to improve efficiency in clinic operations, improve surveillance and management of the disease at a systems level, and ultimately reduce turnaround time to commencement of antiretroviral therapy (ART). For example, the availability of real-time HIV testing data could allow public officials to rapidly identify local outbreaks of the disease and implement a timely and effective public health response.

Africa accounts for 70% of the world’s population living with HIV and close to two-thirds of newly infected individuals [2]. Currently, the region experiences uneven access to HIV tests, long turnaround time of HIV testing, delayed time initiation of ART, and poor retention and adherence with therapy [3]. The high HIV incidences across sub-Saharan Africa mount pressure on decentralized services, which have been underutilized [4,5], in allowing infected individuals to know their status with subsequent linkage to care. Increasing the capability of decentralized testing will be critical in an effort to allocate resources to people and places of greatest need [6,7] to achieve an HIV-free generation (a goal of United Nations sustainable development plans by 2030 [8]).

In Rwanda, detailed household surveys have indicated higher HIV incidences than previously estimated [9] and point to the need for more rapid and detailed characterization of incident infections in planning for an effective national strategy for at-risk populations. HIV incidence in Rwanda seemed to decline after the 1990s with the provision of ART [9,10]. While 160,000 people in Rwanda receive treatment with ART [11], a recent study highlighted the need to understand HIV incidence at a more granular level than currently available in order to reduce HIV infections in the country [9]. More specifically, the study highlights the need for understanding HIV incidence subnationally and within different populations [12], in contrast to using uniform national models for planning HIV programs at local levels that could present many biases [13]. In Rwanda, a relatively low national HIV incidence (compared to other sub-Saharan Africa countries) masks wide variations across groups and demographics [14].

Novel mobile phone technologies are being developed and tested to expand HIV care to decentralized settings [8,15-17]. While some examples include mobile devices and diagnostics to increase adherence to medical appointments [18-21] and to ART therapy [22-28], most mobile health technologies for HIV [29] focus on short message service (SMS) texting. While potentially useful for different aspects of HIV management, these studies did not focus on tracking of HIV test results, let alone doing so with geospatial coordinates provided by mobile phones. Technologies associated with smartphones (ie, mobile phones with enhanced computing power that can run native software programs and can connect to the internet) and mobile phone apps have only been tested recently [30]. Despite the potential of geospatial data on mobile phones, there are few studies on leveraging this information to track HIV incident infections in real time. If such geospatial data could be collected, they could enable HIV test results to be linked to geospatial coordinates. Studies in South Africa and Lesotho found that visualization of georeferenced data (collected by analyzing existing sources of information or by field surveys equipped with Global Positioning System [GPS] receivers, respectively) has the potential to efficiently guide HIV program operations [31,32]. In neither study were the objectives to link geospatial information to HIV test results or to obtain the GPS coordinates using mobile phones.

In this study, we paired a portable reader for interpreting the results of HIV lateral flow tests with a mobile phone app to track HIV test results in urban and rural locations in Rwanda. In a point-of-care setting, a health care worker performs an HIV rapid test. The technology tested in this study first enables the health care worker to use a customized lateral flow reader (LFR) to read the results of the HIV rapid test as positive or negative. Second, the health care worker can instantly record within a mobile app the HIV test result, and the result can be sent instantly or at the next point of internet connection to the cloud. After integration to a relational database stored on the cloud, the results are immediately viewable with geospatial context and in real time by health officials who can allocate resources to local clinic workers efficiently in order to stop HIV outbreaks at their onset. The results from the study aim to lay the foundation for a scalable method to improve the efficiency and quality of identifying HIV incidences quickly in developing countries.

## Methods

### Development and Customization of Lateral Flow Reader Hardware

We purchased 4 ESEQuant LFR readers (Qiagen Inc) for digital interpretation of band intensities in lateral flow tests. The LFR machines consist of 2 parts: main body and drawer. On the main body, the screen and 5 buttons control the program that runs the tests and displays the test results. For the drawer, we designed and manufactured (via a 3D printer) a custom white holder to fit the exact size of an Alere Ab/Ag combo test strip (Abbott) for analysis. The customized LFR can read the control/Ab/Ag lines shown on an Alere Ab/Ag combo test strip and display the results.

**Figure 1:**
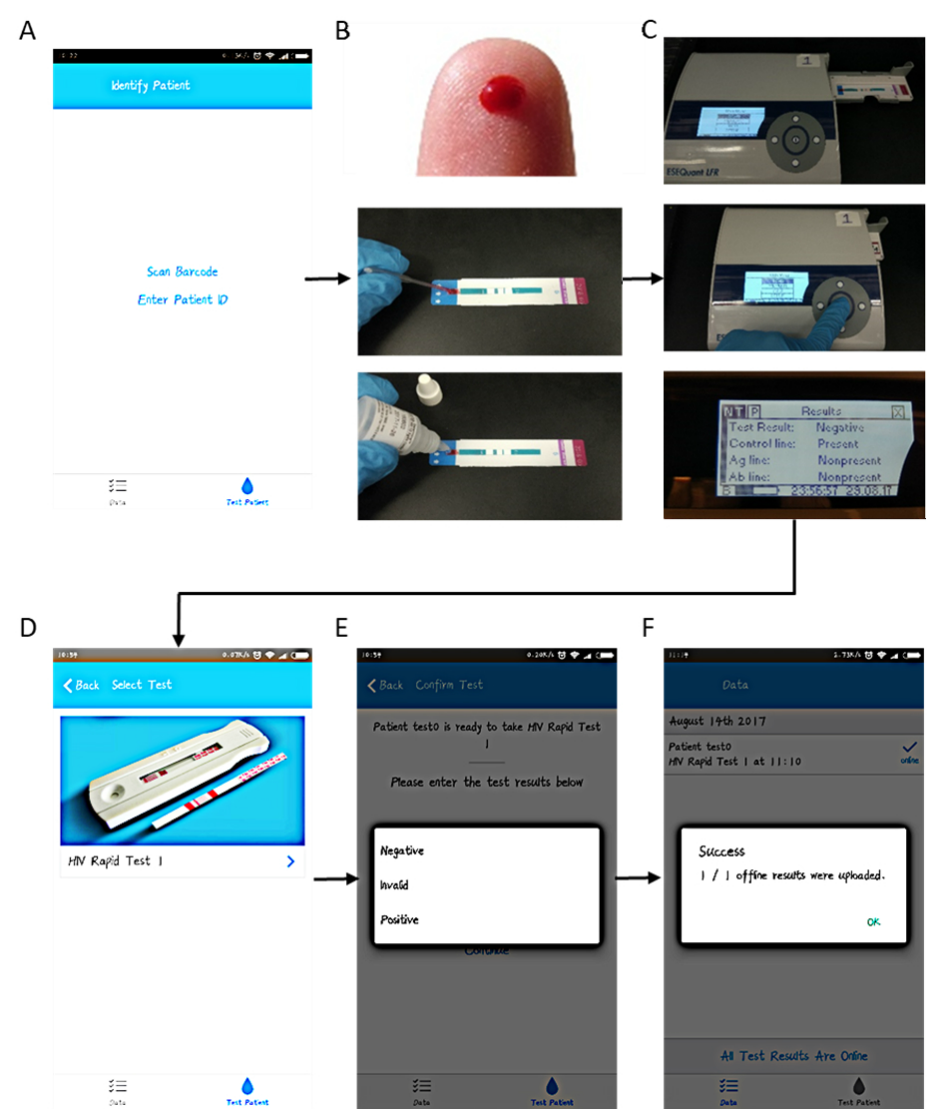
Step-by-step illustration of clinical testing. (A) App instructs user to perform an Alere HIV rapid test. (B) User performs a finger prick and places a drop of blood on the lateral flow strip. (C) The HIV test is placed in a lateral flow test reader, which scans the test and produces a reading. Here, the test result is negative, and the control line is present to indicate a valid test. (D) App displays the HIV rapid test model to be selected. (E) The patient ID and test results are entered into the app. (F) Results are uploaded to the cloud either at the time of test or later when internet is available.

The LFR can either work separately or remotely when connected to a personal computer. In remote mode, several important parameters such as incubation time, scanning positions, detecting range, and detection limitation can be controlled by the software and programmed into the reader. Using lateral flow tests with HIV-positive and HIV-negative samples for calibration, we customized the spatial positions of the 3 stripes of the Ab/Ag/control lines. A built-in peak detection function of the software would determine within the designated spatial positions whether a line would be classified as present. We calibrated all the LFRs with our customized method and provided the readers to the testing sites for use (Figure 1 A-C).

### Design and Coding of Mobile Software

To develop a mobile app to electronically record and transmit test results (Figure 1 D-F), we coded the app by using a cross-platform development tool called React Native. React Native allowed us to port the application, written in Javascript, to both iOS and Android devices (although all mobile phones used in this study were Android) while using platform-specific, native implementations of features such as GPS location and networking.

The mobile app used local storage drivers to save HIV test results to the device in the absence of internet connection. Once a connection was established, test results could be uploaded to our internal PostgreSQL database running on Google’s Cloud Compute platform. PostgreSQL is an open-source relational database with an emphasis on extensibility and standards compliance. As a database server, its primary functions are to store data securely and return that data in response to requests from other software applications. We also added an intermediary Node.js webserver running on Heroku to mediate the communication between the mobile device and database. A single HIV test result contained the following information: patient ID, test ID, result (positive, negative, or invalid), time, latitude, and longitude.

We used Knowi, an online data visualization tool, to view HIV test results and create geographic heatmaps of patient test results. Knowi connected directly to our internal database using read-only database credentials. Knowi enables visualization, warehousing, and reporting automation from PostgreSQL along with other unstructured and structured data sources.

### Ethics Review Approval

The study protocol was approved by the Rwanda National Ethics Committee. Documents on patient consent, health care worker consent, data confidentiality, patient questionnaire, and health care worker questionnaire were approved by the committee. The questionnaire for health care workers collected information on the usability of the technology, while the questionnaire for patients queried the demographics of the patients. In addition, the consent form and questionnaire for patients were translated into Kinyarwanda to facilitate interactions with patients who were not fluent in English.

### Study Setting

The study took place at 3 sites in Rwanda over 4 weeks in February and March 2018. The 2 urban sites in Kigali were Masaka District Hospital (DH) and Kibagabaga DH. One rural site was Kabaya DH in the Ngororero District of the Western Province of Rwanda. Kabaya DH has a capacity of 144 beds and serves 188,902 inhabitants and is geographically difficult to access due to the lack of reliable roads and bridges, especially in the rainy season.

### Recruitment and Training of Health Care Worker Participants

At the 2 urban sites, we invited clinical and laboratory staff to participate in the study. For the 2 sites in Kigali, 4 health care workers in each facility (8 total) participated. In Masaka DH, 3 nurses and 1 lab technician participated (2 male, 2 female). In Kibagabaga DH, 2 nurses, 1 lab scientist, and 1 midwife participated (4 females). At Kabaya, we invited clinical and laboratory staff to participate in the study, and 12 health care workers at Kabaya participated: 5 A1 nurses, 2 A2 nurses, 4 lab technicians, and 1 midwife (8 male, 4 female). (A1 refers to completion of 3 years of postsecondary school, while A2 refers to completion of only secondary school.)

Health care worker participants were trained in the following modules: overview of project (background, aims, and procedure), review of health care worker consent form and data confidentiality agreements, demonstration of LFR, demonstration of mobile app, review of patient consent form (translated) and questionnaires for patients (translated) and health care worker, and review of study plan. At the conclusion of the trial, laboratory and clinical staff were interviewed using the health care provider questionnaire.

### Recruitment of Patients

Patients for the 3 sites came through maternity/gynecology and outpatient departments and were scheduled to be tested for HIV (Alere Determine HIV Combo+ Stat Pak, Abbott Laboratories) through provider-initiated testing. All such adult patients (aged 21 years and older) during the study period were invited by health care workers to enroll. Individual interviews were held in a private space provided by the health facility to protect subject confidentiality. After the study was introduced to the patient, potential participants were informed in their mother tongue about the objectives of the study and the fact that their participation was voluntary. They were informed that they are free to choose not to participate in the study or withdraw at any time with no explanation required, and they will not suffer any negative consequences for their decision. With guidance from health care workers, those who agreed to participate reviewed and signed an informed consent form in Kinyarwanda, their mother tongue, and were provided 1000 RWF (US $1.15) as compensation for their time. Completed consent forms were stored separately from study documents, and names were not recorded on any data documents reviewed in the study.

### Operation of Technology

Health care workers performed the Alere Determine HIV-1/2 combo tests with a finger-pricked patient blood sample. The completed test strip was placed into the customized and precalibrated LFR, and the LFR digitally displayed (unambiguously, as opposed to visual interpretation) a positive or negative result. Results of the HIV tests as visually interpreted were also recorded with pen and paper, and discrepancies relative to the LFR result noted.

Next, the provider input a deidentified patient ID and test result (positive, negative, or invalid) into the mobile app. We purchased locally available mobile phones for the study. The mobile phones were from Impress (Vertex; 60,000 RWF [US $69]). As described previously, the mobile app assists in the registration of patient test results alongside the location of testing down to the street level. The data input by the health care worker, alongside the GPS information, were saved into the phone’s memory. The health care worker either uploaded this information to the cloud database immediately (if internet connectivity was available) or later (when internet connection became available). Internet connectivity, which can be intermittent, was not required for the test results to be recorded.

After each testing procedure, patients were interviewed by the health care worker using the patient questionnaire in Kinyarwanda.

## Results

### User Statistics

After approval of the study protocol by the Rwanda National Ethics Review Committee, we worked with the Directors General of the 3 sites to conduct the trial. Four health care workers at each urban site and 12 at the rural site were trained in the objectives of the trial and the details of the protocol, including issues related to patient consent and confidentiality. From these sites, we enrolled 513 patients at Masaka DH and 596 patients in Kibagabaga DH, for a total of 1109 patients across the 2 sites. For our rural site, we enrolled 1081 patients at Kabaya DH. Remarkably, 100% of eligible patients who were approached agreed to participate at Kabaya DH (similar to the 2 urban sites).

The trial took place over a 4-week period in spring 2018 Table 1. Of the patients whose HIV results were tracked, 91.51% (2004/2190) of the results came with a phone-generated GPS location. (We were also able to manually add the GPS location for the remaining patients since we knew the location of the testing.) The results that did not come with automatic GPS coordinates came primarily earlier in the trial, when the location settings on the phone were not set properly. The problems were mostly resolved after switching “Turn on Location” to on and restarting the phone. Also, a reading of result showed “invalid” if the Alere test was untested, or more likely, if the drawer of the reader was empty. The few invalid results came early in the trial when 3 health care workers did not place the HIV test into the reader or sufficiently firmly press the test down into the housing; after a quick reminder of the procedure during the first 2 weeks, there were no more invalid results. Of the valid tests, the LFR produced the same readings as visual interpretation in 100% of the cases (2166/2166), with 0 discrepancies.

**Table 1:** Summary of the trial data.

Sites	Participants, n	Recordings without GPS^a^, n (%)	Recordings with GPS, n (%)	Invalid recordings, n (%)	Recordings showing positive HIV, n (%)
Masaka	513	62 (12.1)	451 (87.9)	4 (0.8)	39 (6.5)
Kibagabaga	596	32 (5.4)	564 (94.6)	0 (0.0)	9 (1.5)
Kabaya	1081	92 (8.5)	989 (91.5)	20 (1.9)	23 (2.1)
Total	2190	186 (8.5)	2004 (91.5)	24 (1.1)	71 (3.2)

^a^GPS: Global Positioning System.

**Table 2:** Demographics of patients at each site. Questionnaires that did not record a gender or report the testing of HIV were excluded from the analysis.

Characteristics		Masaka DH^a^	Kibagabaga DH	Kabaya DH
Subjects, n		513	596	1081
Questionnaires analyzed (correctly filled out), n		507	593	1057
Female, n (%)		459 (90.5)	593 (84.5)	668 (63.2)
Own mobile phone, n (%)		345 (68.0)	506 (85.3)	755 (71.4)
Own smartphone or internet-enabled phone, n (%)		40 (7.9)	123 (20.7)	78 (7.4)
**Means of transportation to hospital, n (%)**				
	Motorcycle	233 (46.0)	161 (27.2)	29 (2.7)
	Public transportation	93 (18.3)	291 (49.1)	120 (11.4)
	Walk	147 (29.0)	66 (11.1)	889 (84.1)
Time to travel to hospital: less than 2 hours, n (%)		443 (87.4)	551 (92.9)	888 (84.0)
Employed (yes), n (%)		125 (24.7)	178 (30.0)	467 (44.2)
**Annual income, RWF^b^ (USD)**				
	1st quartile	333,000 (383)	500,000 (575)	60,000 (69)
	Median	400,000 (460)	900,000 (1035)	255,000 (296)
	3rd quartile	765,000 (879)	1,200,000 (1379)	716,250 (823)
**Literacy level**				
	Tertiary (A1/A0/Bachelor), n (%)	23 (4.5)	35 (5.9)	58 (5.5)
	Secondary (S1-S6), n (%)	186 (36.7)	256 (43.2)	249 (23.6)
	Primary (P1-P8), n (%)	265 (52.3)	264 (44.5)	311 (29.4)
	Informal (none), n (%)	33 (6.5)	38 (6.4)	439 (41.5)

^a^DH: District Hospital.

^b^RWF: Rwandan franc.

From analysis of a survey (Table 2), across the 2 urban sites, the patients at Kibagabaga DH are higher in median income (χ^2^_1_=39.2, *P*<.001, by the Mood median test), literacy (χ^2^_3_=7.86, *P*=.49), and ownership of mobile phones (χ^2^_1_=45.4, *P*<.001) and smartphones (χ^2^_1_=32.0, *P*<.001). At the rural site, the patients at Kabaya DH consisted of more males than at the urban sites (there was a campaign for male circumcision at the time of the trial). In general, the rural patients were less likely to own mobile phones (χ^2^_1_=11.2, *P*=.001) and smartphones (χ^2^_1_=31.7, *P*<.001), walk to the hospital, and while they were more likely to be employed (χ^2^_1_=62.1, *P*<.001), they had lower median income (χ^2^_1_=35.0, *P*<.001, by the Mood median test) and literacy (χ^2^_3_=376.9, *P*<.001) than those at the 2 urban sites (chi-squared tests comparing the rural site to both of the urban sites combined). For example, 41.53% (439/1057) of patients at Kabaya DH had no formal literacy.

Across all 3 sites, the percentage of patients who own mobile phones was high (at least 68% at each site), but only a smaller percentage (at most 20%) owned mobile phones that could surf the internet.

### Real-Time Geographical Dashboard to Street Resolution

**Figure 2:**
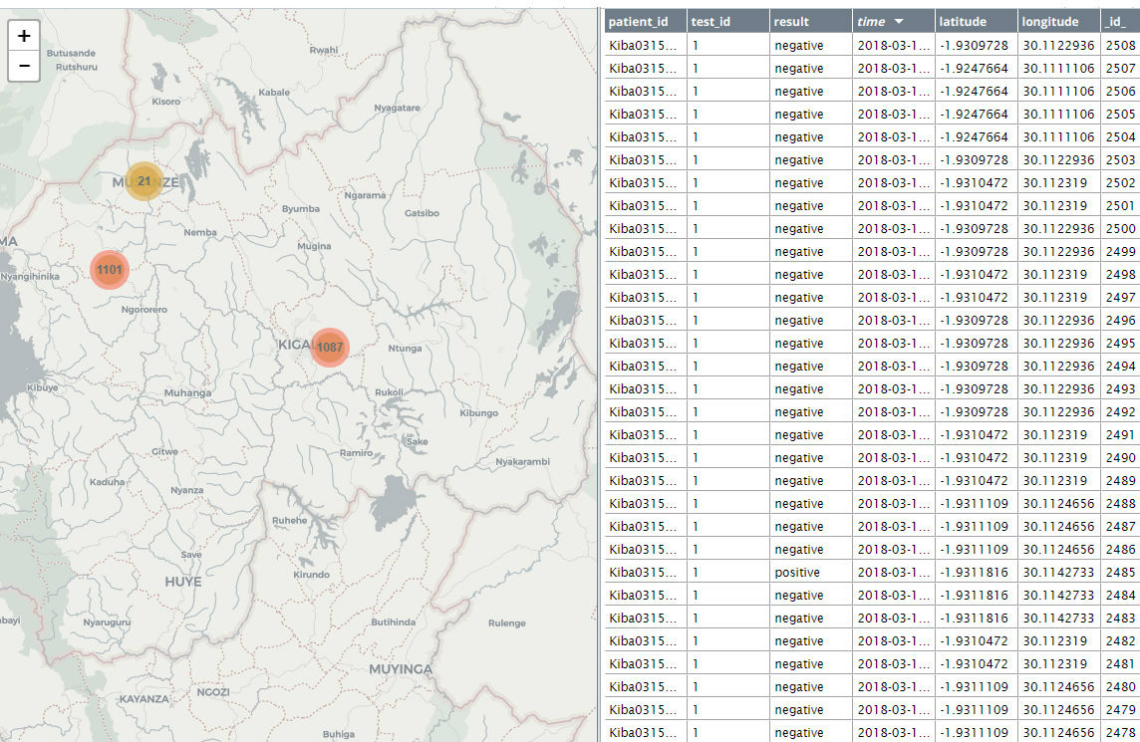
Real-time dashboard of HIV tests tracked in Rwanda. On the map to the left, tests done in Kigali and northwest Rwanda are shown. The right shows the log of the tests as they are recorded.

The mobile app registered each HIV test result. As shown in the map of Rwanda (Figure 2), the results were viewable on the dashboard immediately.

**Figure 3:**
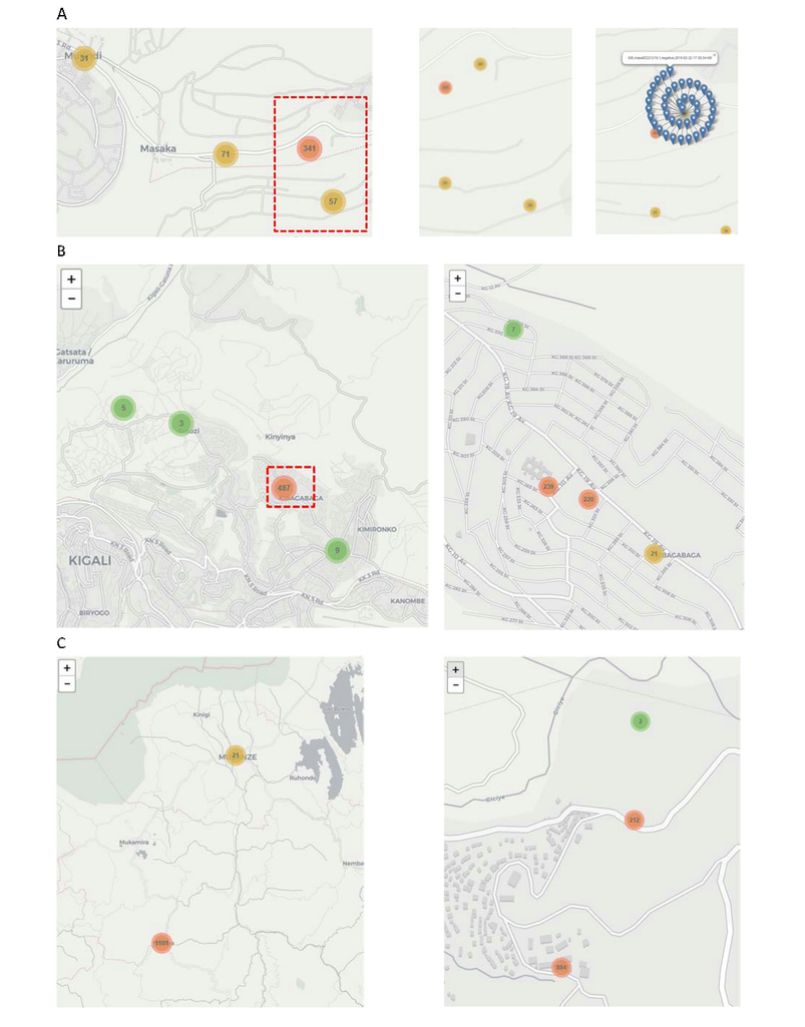
Real-time dashboard of the 3 sites to street resolution. (A) Masaka District Hospital: HIV test results at Masaka District Hospital (left); zoomed region of the red box in left image (middle); clicking on the number 40 showed each of the test results (right). (B) Kibagabaga District Hospital: HIV test results at the site (left) and zoomed image on the red box on the left, showing fine distinction of test locations to street resolution (right). (C) Kabaya District Hospital: HIV test results at the site(left) and xoomed image on the red box on the left, showing fine distinction of test locations to street resolution (right). Colors of each cluster indicate the number of samples (green=1 to 10; yellow=11 to 99; red=100 or above).

As shown in the map, 1087 results were recorded in Kigali and 1122 results in Northwest Rwanda. When zooming into Kigali, one can focus on the 2 sites of Masaka and Kibagabaga separately. First, with Masaka (Figure 3), one can see the HIV test results, including multiple subsites (as performed by different health care workers) at the site, down to street-level resolution. Clicking on 1 of the numbers revealed each of the HIV test results. Similar geographical resolution was achieved with Kibagabaga (Figure 3), showing several test locations as performed by health care workers. In addition, zooming in on the map of Northwest Rwanda showed test results at Kabaya DH to street-level resolution as performed by the 12 health care workers (Figure 3).

### Survey of Health Care Workers

At the end of the trial, we performed surveys of the patients and health care workers. A summary of the results of the survey of health care workers is shown in Table 3.

**Table 3:** Results of survey of health care workers.

Topics		Responses answering yes, n (%)	
**Rapid HIV testing**			
	Were you trained in HIV rapid testing?		20 (100)
	Do you find it difficult to interpret the results of rapid tests?		2 (10)
**Experience with lateral flow reader**			
	Have you used the Junco LFR^a^?		8 (40)
	How many patients with the Junco LFR?		103
	Do you feel the LFR made HIV testing easier?		15 (75)
	Do you feel the LFR made HIV testing faster?		17 (85)
	Do you feel the LFR made HIV testing more difficult?		3 (15)
	Do you feel the LFR made HIV testing slower?		3 (15)
	Would you like to use the LFR again during HIV testing?		16 (80)
	Would you recommend the LFR to others?		14 (70)
**Mobile app**			
	Did you find the mobile app easy to use?		19 (95)
	Would you prefer to use the mobile app over paper records?		19 (95)
	Would you use the mobile app again during HIV testing?		20 (100)
	Would you recommend the app to others?		20 (100)
**Mobile phone**			
	Do you own a mobile phone?		20 (100)
	Do you own a smartphone?		19 (95)

^a^LFR: lateral flow reader.

The 20 health care workers were highly satisfied with the technology. They were most favorable toward the mobile app, finding it easy to use and preferable over paper records. No internet was needed at the time of performing the test (connectivity was required to upload the results, either immediately or later). All of respondents would use the mobile app again during HIV testing and recommend the app to others. While they were provided mobile phones for the trial, 100% of the health care workers owned phones, with 95% (19/20) owning smartphones and using the phones for internet surfing.

The health care workers were slightly less enthusiastic about the LFR. Overall, 80% (16/20) would like to use the LFR again during HIV testing, and 70% (14/20) would recommend it to others. The health care workers at Kabaya were more enthusiastic about the LFR: 83% (10/12) would like to use the LFR again during HIV testing, and 83% (10/12) would recommend it to others.

### Survey of Patients

We also conducted and tabulated the results of a survey of the patients across the 3 sites. Results were recorded by pen and paper and later transcribed into a computer. A summary of the results is shown in Table 4.

Across the 3 sites, 42.7% (253/593) to 71.0% (360/507) of patients received their test results within 30 minutes, with a sizeable percentage (lowest of 26.6% [135/507] at Masaka DH to highest of 48.2% [286/593] at Kibabaga DH) waiting past 30 minutes. At the 2 urban sites, 68.0% (345/507) to 85.3% (506/593) of patients owned cell phones (with most using them for calling, texting, and listening to music). At the rural site, 71.43% (755/1057) of patients owned cell phones (with most using them for calling and texting).

**Table 4:** Results of survey of patients.

Variable/question			Masaka DH^a^	Kibagabaga DH	Kabaya DH
Subjects, n			513	596	1081
Questionnaires analyzed (correctly filled out), n			507	593	1057
**Study subject sex, n (%)**					
	Female		459 (91.5)	501 (84.5)	668 (63.2)
	Male		48 (9.5)	02 (15.5)	389 (36.8)
Have you had a laboratory examination on your blood today? (yes), n (%)			507 (100)	593 (100)	1054 (99.7)
**For which laboratory examinations was your blood drawn today? n (%)**					
	HIV		507 (100)	592 (99.8)	1042 (98.6)
**How long did it take you to get the laboratory results? n (%)**					
	Less than 30 minutes		360 (71.0)	253 (42.7)	681 (64.4)
	30 minutes to 1 hour		70 (13.8)	193 (32.5)	225 (21.3)
	1 to 2 hours		58 (11.4)	63 (10.6)	101 (9.6)
	Over 2 hours		7 (1.4)	30 (5.1)	37 (3.5)
	Not stated		12 (2.4)	54 (9.1)	13 (1.2)
Do you own a mobile phone? n (%)			345 (68.0)	506 (85.3)	755 (71.4)
**If yes, what type? n (%)**					
	Basic phone (text, calling, no internet)		293 (57.8)	408 (68.8)	662 (62.6)
	Smartphone (can download apps) or internet-enabled phone (check email, browse internet)		40 (7.9)	97 (16.4)	69 (6.5)
	Model not specified		12 (2.4)	1 (0.2)	24 (2.3)

^a^DH: District Hospital.

## Discussion

### Principal Findings

We have demonstrated a technology that successfully recorded HIV test results. We paired a portable reader for interpreting the results of HIV lateral flow tests with a mobile phone app to track over 2000 HIV test results in urban and rural locations in Rwanda and could immediately view the HIV test results with geospatial context and in real time. While most health care workers felt the LFR was effective and would use it again for HIV tests, some workers felt it slowed the process. Also, the LFR experienced some operational issues that were resolved within a week. All were satisfied with the mobile app.

The use of mobile phones for HIV diagnostics has so far been limited, with most of the work focused on the outdated SMS messaging technique. There may be a perception that apps require constant internet connectivity and expensive smartphones and are not amenable to aiding HIV diagnostics in developing countries. Our technology does not require constant internet connectivity and makes use of the full power of apps on low-cost (less than $70 USD) smartphones, which over 90% of the health care workers personally own (depending on the demographics). The technique was judged to have high user acceptability, with 100% of the health care workers recommending the app.

While this study was not designed to accurately measure prevalence, we note that the Kigali sites reported 4.3% prevalence, compared to 5.6% in urban population (and 6.1% in Kigali) as previously reported [9]. (The lower apparent prevalence in Kibagabaga DH, being located in Gasabo district, may reflect more patients visiting from rural areas than Masaka DH, located in Kicukiro district.) In our study, the rural site of Kabaya DH reported 2.1% compared to 2.6% as previously reported for rural population [9].

### Limitations

The technology was effective. Overall, 92% of the HIV test results had autogenerated GPS coordinates (with a much higher percentage in the last 3 weeks after the phones were set correctly). The results suggest that this technology can effectively scale (especially if use of an LFR is not required) to the whole country compared to expensive and labor-intensive community cohort–based questionnaires by leveraging the power of mobile phones. However, pointing to the limitations of this study, several important steps still need to be addressed before significant public health impact can be achieved: patient records will need to be integrated with existing electronic health record systems before such a technology can replace (rather than complement) current patient records, and replacement of the functions of the LFR with the app could streamline workflow and increase usability. Also, the reliance on manual entry of the data could still introduce errors, although currently the LFR keeps a backup log of the results (so the results can be cross-checked using the time stamp), and in the future, a picture of the rapid test will be taken and kept on record for cross-validation of results. Finally, to increase the success rate of using the technology, including among users of different levels of education and technical proficiencies, we could ask for a successful skills demonstration after the training and before starting the trial.

### Conclusions

Toward the Joint United Nations Programme on HIV/AIDS 90-90-90 targets for HIV patients and diagnostics, we tested a mobile phone–based technology for tracking HIV incidences in Western Rwanda and at rural locations, where unexpected incidences emerged [9]. In rural settings, the LFR was perceived to work faster compared to the existing workflow (100% in rural sites to 63% urban sites) and was recommended more highly (83% rural sites to 50% urban sites). The app was uniformly praised for its speed of use and effectiveness, garnering 100% recommendation.

For the way forward, we are buoyed by the effectiveness of our technique and the uniform enthusiasm especially for the app (100% enthusiasm from all 20 health care workers). We plan to expand a version of the app that would obviate the need for an LFR, which could improve the scalability of the method to improve public health surveillance of HIV and other infectious diseases. The results from the study aim to lay the foundation for a scalable method to improve the efficiency and quality of identifying HIV incidences quickly in developing countries. In the future, this technology could also be applied to HIV home testing, with 10% of our surveyed patients already owning compatible mobile phones. We will work to scale this technology in Rwanda and beyond, which, at low marginal cost, leverages the power of mobile phones to track HIV incidences in real time and with proper spatial context.

## Abbreviations

ART: antiretroviral therapy
DH: District Hospital
GPS: Global Positioning System
LFR: lateral flow reader
SMS: short message service
